# Maternal Competence, Maternal Burnout and Personality Traits in Italian Mothers after the First COVID-19 Lockdown

**DOI:** 10.3390/ijerph19169791

**Published:** 2022-08-09

**Authors:** Concetta Polizzi, Giulia Giordano, Sofia Burgio, Gioacchino Lavanco, Marianna Alesi

**Affiliations:** Department of Psychology, Educational Science and Human Movement, University of Palermo, 90128 Palermo, Italy

**Keywords:** psychological burn-out, competence, neuroticism, COVID-19

## Abstract

This study aimed to investigate the maternal sense of competence and maternal burnout in Italian mothers during the COVID-19 pandemic. The sample was composed of 278 mothers of children/adolescents aged 4 to 17 years old. Participants were recruited after the end of the first spring total Italian lockdown (June–October 2020) through online advertisements on websites and social media. We hypothesized a model in which a specific personality trait, such as neuroticism, affected maternal competence by the mediating role of maternal burnout. Results showed that neuroticism was directly and negatively predictive of perception of maternal competence, and it was negatively associated with maternal burnout, specifically specific antecedents that were strictly related to parental burnout. ANOVA results highlighted that the maternal level of education affected maternal competence in terms of satisfaction. In contrast, the working regimen during the first lockdown for COVID-19 affected maternal competence in terms of efficacy. Maternal burnout was affected by atypical child development in terms of both common (job burnout, stress management abilities) and specific (parental burnout) antecedents. COVID-19 strongly increased the risk of maternal burnout, resulting in mothers having a poor perception of their own competency. This is particularly the case in the presence of a neurotic personality.

## 1. Introduction

During the first wave of the COVID-19 pandemic (from 10 March to 17 May 2020), the Italian Government adopted strict measures of social restriction to limit the contagion of the infection. Severe measures ranged from home confinement and home working, to the closure of non-essential businesses and the closure of all schools, because there were no guidelines or regulatory indications that were useful for facing the crisis and supporting people’s resilience.

When borders between work and a home broke down, parents were forced to face several pressing concerns and to reorganize their schedules to satisfy new exceptional and challenging demands in several domains, which are defined by: work, parenting, family relationships, and household commitment [1,2].

Previous research highlighted the risk factors that can enhance parental stress: having a personality profile oriented to neuroticism, being a mother, having an age range between 18 and 30 years, having a child with special needs, being a single parent, working part-time or having no professional occupation, having a previous history of trauma or disease, having an infected relative [3,4,5].

In a specific manner, personality traits can influence the perception of threats and lead to pandemic responses [6]. Parents with personality profiles characterized by neuroticism, worrying, emotional variability, and insecurity experienced higher levels of distress and negative emotions [3]. Caci and collaegues [7] found that people with higher levels of neuroticism expressed major fears about the impact of COVID-19 on daily life, and were more bored and involved in negative fantasies and thoughts. The Five Factors Model (FFM) describes neuroticism as the most crucial trait for facing fears. Individuals with high neurotic traits are typically characterized by an increased perception of risk, greater emotional reactivity, more sensitivity to punishment signals, and fewer resources to manage and cope with stressful events.

Increasing research has highlighted how mothers are at higher risk of psychological stress than fathers [8,9,10]. This is because women are forced to balance many work and family responsibilities, from working remotely to taking care of children, supervising home education, and homeschooling school-aged children [11,12]. Moreover, mothers who perceived higher rates of parenting stress were more likely to attribute more negative emotions to their children, to be less sensitive to their needs, and to have more maladaptive and undesirable interactions with them [13].

Under the condition of heavy emotional stress caused by COVID 19 confinement, high levels of parental stress spilled over into the state of parental burnout [14]. Parental burnout develops when parental resources are insufficient to cope with the risk factors, and significantly increase the levels of parental stress that outweigh resources and protection factors [15,16]. Parental burnout cannot be interpreted as ordinary parental stress; rather, it is a context-specific syndrome resulting from enduring exposure to chronic parenting stress [15,16,17]. It has four components: emotional exhaustion, saturation, contrast, and emotional distancing [18]. The first refers to a lack of energy and weakness; the second to a state of indifference and apathy in sharing time with children; the third to a condition of indifference towards one’s role as a parent; and, finally, the fourth to emotional distance from children. Consequently, the first symptom of parental burnout is overwhelming exhaustion related to one’s parental role. Parents feel tired when getting up in the morning and having to face another day with their children; they feel emotionally drained by the parental role to the extent of thinking about their role. Moreover, exhausted parents become less involved in parenting and the relationship with their children, and interactions are limited to functional and instrumental aspects at the expense of emotional elements. Another symptom is a loss of accomplishment in one’s parental role: parents feel fed up with parenting, can no longer stand their role as a father or mother, and no longer enjoy being with their children. Notably, all these symptoms and states contrast with how the parent previously felt before parenting [18]. Mikolajczak and colleagues [15] found that parents with burnout showed a sense of incompetency in the parental role, combined with a loss of confidence in their judgment and parenting skills. Significant responsibilities of the parental role and elevated social expectations can decrease the trust and the sense of parental competence, especially in prolonged distress conditions [19,20]. Several dispositional factors related to parental burnout have been highlighted, such as being female and a younger parent, children’s age, job, financial insecurity, and limited social support [2]. In addition, personality traits play a vital role among these factors because they moderate the vulnerability to parental burnout [21]. Among these traits, in particular, neuroticism is considered the major risk factor [2,15].

The purpose of the present online cross-sectional survey was to investigate maternal burnout related to the management of parental functions, neuroticism, and sense of parental competence in Italian mothers during the COVID-19 pandemic, specifically following the first total quarantine.

The Balances Between Risks and Resources framework was adopted [15]. This model of parental burnout postulates an imbalance between parental risks and protective factors, and discriminates between specific and common risk factors. The former predict parental burnout and include high parental standards, poor parenting practices, and poor co-parenting. At the same time, common risk factors equally contribute to predicting parental and job burnout, and concern a perfectionist personality, poor stress management abilities, and pessimistic tendencies. Mothers with mostly common risk factors in their balance are vulnerable to both forms of burnout, whereas mothers with specific risk factors are vulnerable to parental burnout.

Compared to other research that has investigated specific risk factors for mothers’ stressful conditions and mental health symptoms, such as depression and anxiety [22,23,24,25], the present study provides added value because it focused on mothers’ personalities. This study investigated the possible influence of the personality traits of mothers and, in particular, neuroticism, on the perception of maternal competence, with the mediating role of maternal burnout. Many studies have investigated the role of various risk factors for the parents’ well-being, such as psychological distress, lower resilience, lower number of perceived social connections, being single, having a child with special needs, having many children or having young children, the perception of the difficulty of quarantine, etc. [3,26,27]; however, only in rare cases has the role of dispositional variables been investigated, such as the mother’s personality profile, and the possible sense of parental competence perceived by the mothers.

The following goals were investigated:

Goal 1: The maternal sense of competence and maternal burnout, following the first total COVID-19 lockdown, may have been influenced by the specific variables of mothers and/or children. Therefore, we hypothesized that the following factors may have contributed to decreasing the maternal sense of competence and increasing the levels of maternal burnout: (1) age, level of education, working condition during the lockdown (regarding the mothers); (2) age, sex, and presence/absence of a disability or special needs (regarding the children).

Goal 2: The maternal sense of competence and maternal burnout may have been negatively correlated following the first total COVID-19 lockdown. We hypothesized that mothers with a higher level of burnout would show a lower maternal sense of competence.

Goal 3: The relationship between mothers’ neuroticism and maternal competence may have been mediated by maternal burnout and moderated by specific maternal variables. We hypothesized a possible moderating effect of maternal age in the direct relationship between neuroticism and the sense of maternal competence, and in the relationship between specific antecedents of maternal burnout and maternal competence.

## 2. Materials and Methods

### 2.1. Sample

The sample was composed of 278 mothers of children/adolescents aged 4 to 17, recruited after the end of the first spring total Italian lockdown (from June 2020 to October 2020) through online advertisements on websites and social media (Facebook and WhatsApp), and through a link that led to the questionnaires on Google Forms.

In particular, advertisements on websites could be found on researchers’ personal university pages. Moreover, the snowball sampling method was used among students and researchers.

### 2.2. Measures

All tools were transferred in an online format through Google Forms.

This online survey was administered from June to October 2020. Participants were recruited through online website advertisements by a snowball sampling strategy.

The first page of the survey introduced the aims and the procedures of the study and required participants informed consent before filling in the questionnaires. The participation was voluntary and anonymous. The survey took about 20–25 min to be completed. The study was conducted following the Declaration of Helsinki and was approved by the Bioethics Committee of the University of Palermo (n. 13/2020).

The survey included the following measures. The first was a socio-demographic questionnaire to collect data such as sex, age, nationality, region, marital status, level of education, child’s sex, child’s disorders or disabilities, work regimen before and during COVID-19 pandemic, habits before and during COVID-19 pandemic, social relations re-established after the COVID-19 emergency, use of social networks, support need during COVID-19 pandemic, presence of discomfort during COVID-19 emergency, and ideas relating to the process of the pandemic.

The Balance between Risks and Resources (BR2; [15]) is a self-report questionnaire used to evaluate the maternal burnout perception through two types of antecedent items: the common risk factors, indicating aspects that together constitute equal predictors of job and parental burnout (e.g., “It is difficult for me to reconcile my family life and my professional life”); and the specific risk factors, showing aspects strictly related to parental burnout (e.g., “Due to my parenting responsibilities, I can never find time for myself”).

In particular, the BR2 is composed of 39 bipolar rating items, in which mothers expressed their agreement or disagreement concerning a scale of values ranging from −5 to +5. The total score ranged between −195 and +195. In particular, the common antecedent subscale ranged between −70 and +70; and the specific antecedents subscale ranged between −125 and +125. A positive score showed the presence of resources in parents, whereas a negative score indicated the risks of parental burnout; scores of 0 showed equal levels of risks and resources [15]. The internal consistency was around 0.70. The reliability values were of α = 0.96 for the global scale, α = 0.89 for the common antecedent subscale, and α = 0.94 for the specific antecedent subscale. The original version was translated and adapted to the Italian context with the author’s permission.

The Parenting Sense of Competence-Efficacy Scale (PSOC-Efficacy; [28]) was administered in its Italian version (Questionario del Senso di competenza dei genitori) [29] to measure the efficacy and satisfaction perceived by mothers about their parental role. This self-report questionnaire is composed of 16 items on a 6-point Likert scale (from 1 = strongly agree to 6 = strongly disagree), in which 9 items belong to the “Satisfaction” construct (e.g., “Being a parent makes me tense and anxious” and 7 items belong to the “Efficacy” construct (e.g., “I can find answers to my son’s problems”). 

The Personality Inventory (PI; [30]) is self-report questionnaire composed of 20 items to evaluate personality factors according to the Big Five model [31]. The questionnaire has five subscales: extroversion defined by a search for aggregation, assertiveness, positive emotionality, search for excitement; conscientiousness as the sense of duty and self-discipline; openness concerning experiences and intellectual curiosity; agreeableness as trust in others and ability to cooperate; and neuroticism as a tendency to emotional instability. In particular, for the current study, we considered only the neuroticism subscale. Each item was scored on a 5-point scale with anchors from 1 = strongly disagree to 5 = strongly agree.

### 2.3. Statistical Analysis

As a preliminary step, we derived descriptive statistics (means, standard deviations, and ANOVAs) for mother’s age, level of education, work regimen during the first lockdown for COVID-19, child age, child sex, child atypical development, maternal competence, and parental burnout. Then, in a second step, we computed Pearson correlations for study variables (maternal neuroticism, -br2 total score, specific antecedents, common antecedents, the parental sense of competence total score, satisfaction, and efficacy).

Finally, a mediation moderate model was hypothesized in which we investigated the impact of neuroticism on the parental sense of competence through the common and specific antecedents. We specified maternal burnout as a common and specific antecedent. Mother’s age was used as a moderator on the direct relation between neuroticism and perception of maternal competence, and on the indirect effect of specific antecedents on perception of maternal competence. Mediation analyses were carried out using the SPSS macro, PROCESS. The mediating effect was defined using a bootstrap sample with a 95% confidence level for all confidence intervals.

Analyses were undertaken using SPSS (IBM SPSS Statistics for Windows, Version 26.0. IBM Corp., Armonk, NY, USA).

## 3. Results

### 3.1. Participants

The sample appeared to be highly heterogeneous in terms of the age of respondents (range from 21 to over 50), for which the following seven age ranges were identified, 21–25 years (1.4%), 26–30 years (1.8%), 31–35 years (13.7%), 36–40 years (21.6%), 41–45 years (32.7%), 46–50 years (21.9%), beyond 50 years (6.8%). Most mothers were Italian (98.9%) and married (84.2%), and a smaller percentage were separated (6.8%) or cohabiting (7.9%). Only 1.1% of mothers were single.

Regarding the level of education, the most common qualification (41%) was the degree, then the high school diploma (29.1%). A minority of mothers had a Ph.D. or a specialization (16.2%), a professional diploma (6.8%), and a middle school diploma (6.1%). Only two mothers had elementary school certificates (0.7%).

A share of 34.5% of the sample affirmed they were employees, while 19.1% were freelancers, 15.5% were teachers, and 11.9% were housewives. Furthermore, during the first lockdown caused by the COVID-19 pandemic, most of these professions were practiced in smart working modality (54.4%); the share of mothers who continued to work at the workplace was 22.2%, while a small number of mothers declared they did not work due to job suspension (14.2%) or layoffs (5%), or because they lost their job (4.2%).

Most children were boys (56.1%) and 43.9% were girls; the average age of children was 9.77 years old (SD = 4.05). A share of 86.3% of the children had typical development while 13.7% had special needs such as Autism Spectrum Disorder, ADHD, genetic syndrome, or intellectual disabilities. Only 8.3% of children were born prematurely.

### 3.2. Maternal Competence and Burnout

Means, SDs, and ANOVAs for maternal competence and burnout are shown in Table 1 and Table 2. ANOVA results highlight that the maternal level of education affected maternal competence only in terms of satisfaction [F (2, 275) = 2.179, *p* = 0.05, *ηp*^2^ = 0.039]; while working regimen during the first lockdown for COVID-19 affected maternal competence in terms of efficacy [F (2, 275) = 2.84, *p* = 0.02, *ηp*^2^ = 0.046]. No significant differences were found for maternal age. As regard child variables, only child sex had a significant effect on total score of maternal competence [F (2, 275) = 5.86, *p* = 0.02, *ηp*^2^ = 0.021] and efficacy [F (2, 275) = 10.41, *p* = 0.001, *ηp*^2^ = 0.036]. No significant differences were found for child age and the presence of disability or special needs.

Parental burnout was only affected by child atypical development [F (2, 275) = 8.68, *p* = 0.003, *ηp*^2^ = 0.031]; this was also the case in terms of common antecedents [F (2, 275) = 11.03, *p* = 0.001, *ηp*^2^ = 0.038] and specific antecedents [F (2, 275) = 6.408, *p* = 0.01, *ηp*^2^ = 0.02]. No significant differences were found for mother age, level of education, working regimen during the first lockdown for COVID-19, child age, or child sex.

### 3.3. Correlations between Maternal Neuroticism and Burnout

Correlation results are shown in Table 3; neuroticism was negatively associated with the Balance between Risks and Resources (r = −0.281, *p* = 0.000), and particularly common antecedents (r = −0.389, *p* = 0.000) and specific antecedents (r = −0.201, *p* = 0.001); and with parental sense of competence (r = −0.209, *p* = 0.000), in particular satisfaction (r = −0.197, *p* = 0.001) and efficacy (r = −0.138, *p* = 0.021). Common antecedents were positively associated with parental sense of competence (r = 0.299, *p* = 0.000), in particular satisfaction (r = 0.291, *p* = 0.000) and efficacy (r = 0.184, *p* = 0.002). Specific antecedents were positively associated with parental sense of competence (r = 0.353, *p* = 0.000), in particular satisfaction (r = 0.303, *p* = 0.000) and efficacy (r = 0.272, *p* = 0.000).

### 3.4. Mediation Model

**Figure 1:**
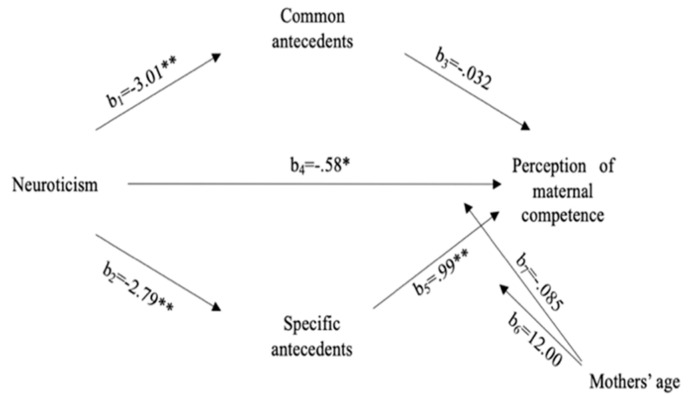
Mediation model. Standardized effect coefficients are reported. Common antecedent effects on perception of maternal competence were not significant. * *p* < 0.05, ** *p* < 0.01.

Results from the mediation model (see Figure 1) highlighted that neuroticism was directly and negatively predictive of perception of maternal competence (b = −0.58, *p* < 0.05), and it was negatively associated with specific antecedents (b = −0.79, *p* < 0.01), which in turn were positively related to the perception of maternal competence (b = 0.99, *p* < 0.01). The level of confidence for bootstrapping showed the significant indirect effect of specific antecedents on the perception of maternal competence (b = −0.2783, 95% CI [−0.5400,−0.0702]). These results highlight that specific antecedents partially mediated the relationship between neuroticism and perception of maternal competence. Neuroticism was negatively associated with common antecedents (b = −3.01, *p* < 0.01), for which no significant association with perception of maternal competence was found.

Moderated mediation models were analyzed using Model 15 to assess the conditional indirect effect of mother’s age as a moderator; it was not significant (b = −2.733, *p* = 0.1187). Furthermore, the effect of mother’s age was not significant for the direct effect of neuroticism on perception of maternal competence (b = 0.0246, 95% CI [−0.0475, 0.1060]). 

## 4. Discussion

The study investigated maternal burnout and the sense of maternal competence, considering the possible influence of personality traits and the moderating effect of specific dispositional factors, such as the mother’s age.

Although the pandemic constituted a risk condition for both parents, in this study we focused exclusively on mothers, assuming that they suffered more from managing their parental role with a significant overload linked to the duty to combine work, child caring, and housekeeping [13,32,33]. Tchimtchoua Tamo [33] analyzed the relationship between mother’s stress (PSI-SF) and their children during the COVID-19 pandemic confinement and concluded that “mothers had more feelings of inability, melancholy, incompetence, and sickness to cope with parenting difficulties than ordinarily”.

Tavares et al. compared depression, anxiety, perceived stress, and social support need of mothers and fathers. They found that mothers showed higher levels of depression and social support than fathers [34].

The first goal was to investigate the influence of mothers’ and children’s variables on the perception of maternal competence and maternal burnout. Regarding maternal competence, significant differences were found for the level of education, the work regimen, and the child’s sex. Mothers with professional and higher levels of education were more satisfied in their parenting role, whereas mothers with work regimens using smart working or subject to layoffs felt a lower sense of parenting efficacy. These results are well documented in the recent literature showing how mothers’ lower education, lower income level, and being unemployed were reported to be related to lower levels of mental health [10,33].

An unexpected result was that the mothers of boys showed the highest perception of maternal competence and efficacy. Regarding parents, the literature has usually described sex as a dispositional factor for parental burnout and demonstrated how mothers were more vulnerable [2]. Our results can be explained by consolidated research that showed that trauma-exposed girls had the greatest probability of internalizing disorders and self-blaming behaviors compared to boys [35].

With regard to maternal burnout, being a mother during the COVID-19 pandemic represented a particular stress condition [22,36], because mothers had to manage several duties linked to different fields, such as parenting, family relationships, work, and housework [1,2]. This situation was exacerbated in the case of a child with atypical development, because of a lack of resources and a complementary prevalence of risk factors. This was found to be a source of burnout related both to the common and specific antecedents. Another study [37] highlighted that home confinement enhanced the stress of parents who have children with a disability or developmental fragility; the sudden and unexpected changes in family routines and lifestyle caused by the COVID 19 pandemic have been experienced as more challenging for children with atypical development and their families given the decrease or temporary interruption in professional support delivered by specialists (physicians, therapists, psychologists, etc.).

Urizar et al. (2022) [38] found that around 32 and 71% of parents of children with disabilities experienced stress conditions, in particular higher levels of depression and anxiety and maladaptive coping strategies. Consequently, these parents, who started from a poor condition, faced several new care problems and were forced to reorganize their daily activities to adapt to their children’s needs [39].

Our second goal was to investigate the relationship between maternal competence and maternal burnout. More distressed mothers, indicating a lack of resources or a prevalence of risk factors, also felt less satisfied and less effective concerning their parental role. During the pandemic, mothers have had to split their time between child care and working at home, while sometimes also experiencing important financial and health concerns, in a condition of substantial social isolation [40]. All of these factors have intensified mothers’ tiredness and frustration, with consequent difficulties in implementing their parenting functions of caregiving and support [37]. Italian women, in particular, suffered from an excess of housework and childcare because Italy is characterized by traditional and conservative gender roles, which aim at assigning housework and childcare to women and paid work to men. Consequently, during the quarantine, 68% of Italian women were found to spend more time in housework and 61% in childcare, compared to 40 and 51%, respectively, for men [41].

As a third goal, we explored the weight of the personality profile of mothers, based on the level of neuroticism, on parental stress during the pandemic. In line with other studies [2,7], which showed that specific personal variables played an essential role in managing the risk condition caused by the pandemic, we found that neuroticism was a direct predictor of the sense of maternal competence and this effect was mediated by maternal burnout as the specific antecedent. High scores on the neuroticism scale were correlated with low scores on the parental burnout scale and, therefore, with a lack of resources, which led to generally high levels of stress and anxiety [42]. This result confirms that people with neurotic personalities are more likely to lack the resources required to manage stress [43]. The highly unpredictable nature of the pandemic increased in mothers having neurotic personalities, a sense of insecurity, and a tendency to generalize fears. These mothers experienced more negative emotions and, in a situation characterized by a total lack of control of events, such as the COVID-19 pandemic, developed more fears about themselves, their children, and their working life than under normal living conditions. Conversely, it has been pointed out that neuroticism is crucial in coping with the COVID-19 pandemic [7]. This personality trait is positively associated with the fear of COVID-19, and with other fears such as the fear of pain [44], the fear of loss [45], and the fear of death [46].

### 4.1. Psychological Support Services

Access to and use of psychological services is crucial to prevent long-term COVID-19 impacts on individuals and their family well-being. Likely as a result of COVID-19-related measures (e.g., physical distancing, shuttering of businesses), individuals primarily endorsed seeking information through online means and phone apps [22]. In the context of the pandemic, online services are a viable option for families [47]. In this sense, the offer of psychological support for parental competence, even remotely, assumes particular importance [37,48,49]. Specifically, psychological support services should be planned to help parents reduce stress levels and the risk of parental burnout, which can be dangerous for parents’ health and the development of children.

### 4.2. Strengths and Limitations

The results of this study should be interpreted with reference to both strengths and limitations. A strength was clarifying the variables that have a more significant impact on mothers’ stress. Another strength can be found in focusing on the role of personality traits in determining the psychological condition of people. Regarding the limitations of this study, the absence of a longitudinal perspective should be emphasized; it would have been interesting to compare these data with those from a different period of the pandemic. Using a transversal rather than a longitudinal design, we will again conduct this survey on maternal stress, perceived sense of parental competence, and personality traits due to the participants’ anonymity. Furthermore, in the current period of the so-called fourth wave, we will verify if the prolongation of a condition of concern for possible infections and the fear of returning to living with further restrictions may have resulted in a further increase in the risk of maternal burnout. An additional limitation concerns the small size of our sample, which was due to the short period taken into account.

## 5. Conclusions

The results of this study suggest that the first total lockdown caused by COVID-19 strongly increased the risk of maternal burnout, resulting in mothers having a poor perception of their own competency. This was particularly the case in the presence of a neurotic personality, demonstrating how many dispositional variables, and especially personality traits, played a more fundamental role than contextual variables.

Future research and mental health interventions should continue to consider and identify specific populations, such as mothers, who appear particularly vulnerable to the psychological implications of the pandemic.

Furthermore, this study allows us to reflect on the role played by the online psychological support services under the conditions of a health emergency, which can foster wider participation. It should also be remembered that psychological support is among the primary needs for which provision must be guaranteed.

## Figures and Tables

**Table 1:** Means, standard deviations, and ANOVAs of the dependent variables (mother age, level of education, work regimen during the first Lockdown for COVID-19, child age, child sex and child atypical development) for the dependent variable maternal competence.

Maternal Competence						
	Total		Satisfaction		Efficacy	
Mother Age	M (SD)		M (SD)		M (SD)	
<36 (*n* = 47)	62.89 (11.16)	F = 1.68 *p* = 0.18 *ηp*^2^ = 0.012	35.68 (7.08)	F = 1.30 *p* = 0.27 *ηp*^2^ = 0.009	26.20 (8.44)	F = 2.58 *p* = 0.07 *ηp*^2^ = 0.018
36–45 (*n* = 151)	59.98 (11.41)		33.70 (7.80)		26.08 (5.82)	
>45 (*n* = 80)	59.42 (9.31)		34.47 (7.06)		25.35 (5.21)	
Level of education						
Primary School (*n* = 2)	59.00 (0.00)	F = 2.05 *p* = 0.06 *ηp*^2^ = 0.037	30.00 (1.41)	F = 2.179 *p* = 0.05 * ηp^2^ = 0.039	29.00 (1.41)	F = 11.21 *p* = 0.30 *ηp*^2^ = 0.022
Middle School (*n* = 17)	60.94 (7.68)		34.24 (4.22)		26.71 (5.91)	
Professional School (*n* = 19)	64.79 (11.09)		36.63 (5.77)		28.16 (6.94)	
High School (*n* = 81)	61.02 (11.35)		34.51 (7.40)		26.52 (5.66)	
Degree (*n* = 114)	60.61 (10.01)		34.94 (7.78)		25.67 (5.58)	
PhD/ Specialization (*n* = 45)	56.22 (12.14)		31.29 (7.98)		24.93 (5.63)	
Work Regimen during the First Lockdown for COVID-19						
Smart Working (*n* = 130)	60.86 (10.03)	F = 1.772 *p* = 0.13 *ηp*^2^ = 0.029	33.95 (7.32)	F = 0.965 *p* = 0.42 *ηp*^2^ = 0.016	25.17 (5.69)	F = 2.84 *p* = 0.02 * ηp^2^ = 0.046
Work at the Workplace (*n* = 53)	60.58 (11.06)		34.34 (7.34)		26.25 (5.52)	
Suspension of Activities (*n* = 34)	60.38 (11.34)		33.38 (8.15)		27.00 (5.66)	
Layoffs (*n* = 12)	60.58 (12.13)		36.42 (7.44)		24.17 (5.73)	
Lost Job (*n* = 10)	68.30 (13.62)		37.80 (9.39)		30.50 (5.58)	
Child Age						
4–6 (*n* = 69)	59.12 (10.77)	F = 1.068 *p* = 0.36 *ηp*^2^ = 0.012	32.91 (7.60)	F = 1.06 *p* = 0.36 *ηp*^2^ = 0.011	26.20 (5.79)	F = 1.67 *p* = 0.17 *ηp*^2^ = 0.018
7–10 (*n* = 93)	61.72 (11.50)		34.83 (6.92)		26.89 (6.27)	
11–13 (*n* = 64)	60.61 (10.56)		34.86 (8.58)		25.75 (4.98)	
14–17 (*n* =52)	59.02 (9.93)				24.73 (5.38)	
Child Sex						
Boys (*n* = 156)	61.69 (10.93)	F = 5.86 *p* = 0.02 * ηp^2^ = 0.021	34.67 (7.53)	F = 1.08 *p* = 0.29 *ηp*^2^ = 0.004	27.02 (5.39)	F = 10.41 *p* = 0.001 ** ηp^2^ = 0.036
Girls (*n* = 122)	58.55 (10.49)		33.73 (7.41)		24.82 (5.93)	
Child Atypical Development						
Yes (*n* = 38) Not (*n* = 240)	59.32 (10.78) 60.47 (10.85)	F = 0.372 *p* = 0.54 *ηp*^2^ = 0.001	34.08 (6.89) 34.29 (7.58)	F = 0.25 *p* = 0.87 *ηp*^2^ = 0.000	25.24 (5.54) 26.18 (5.76)	F = 0.893 *p* = 0.34 *ηp*^2^ = 0.003

Note: *: *p* < 0.05; **: *p* < 0.001.

**Table 2:** Means, standard deviations, and ANOVAs of the dependent variables (mother age, level of education, work regimen during the first Lockdown for COVID-19, child age, child sex and child atypical development) for the dependent variable parental burnout.

Parental Burnout						
	Total		Common Antecedents		Specific Antecedents	
Mother Age	M (SD)		M (SD)		M (SD)	
<36 (*n* = 50)	57.68 (61.01)	F = 0.68 *p* = 0.50 *ηp*^2^ = 0.005	16.83 (20.95)	F = 1.18 *p* = 0.30 *ηp*^2^ = 0.009	40.85 (42.75)	F = 0.394 *p* = 0.67 *ηp*^2^ = 0.003
36–45 (*n* = 163)	57.54 (59.75)		16.53 (23.01)		41.01 (39.96)	
>45 (*n* = 95)	67.06 (63.70)		21.26 (23.69)		45.80 (42.18)	
Level of education						
Primary School (*n* = 2)	58.50 (57.28)	F = 0.773 *p* = 0.57 *ηp*^2^ = 0.014	16.50 (21.92)	F = 0.523 *p* = 0.75 *ηp*^2^ = 0.010	42.00 (35.35)	F = 0.882 *p* = 0.49 *ηp*^2^ = 0.016
Middle School (*n* = 17)	44.65 (59.11)		11.76 (18.38)		32.88 (42.99)	
Professional School (*n* = 19)	75.26 (52.28)		20.21 (18.10)		55.05 (37.20)	
High School (*n* = 81)	57.51 (58.95)		17.15 (22.76)		40.36 (40.60)	
Degree (*n* = 114)	65.20 (61.42)		19.84 (23.06)		45.36 (40.53)	
PhD/ Specialization (*n* = 45)	52.60 (68.31)		16.00 (26.31)		36.60 (43.91)	
Work Regimen during the First Lockdown for COVID-19						
Smart Working (*n* = 130)	60.86 (59.29)	F = 0.476 *p* = 0.75 *ηp*^2^ = 0.008	18.77 (21.83)	F = 0.571 *p* = 0.68 *ηp*^2^ = 0.010	42.09 (40.02)	F = 0.538 *p* = 0.70 *ηp*^2^ = 0.009
Work at the Workplace (*n* = 53)	52.92 (58.31)		14.74 (22.89)		38.19 (38.78)	
Suspension of Activities (*n* = 34)	61.15 (69.49)		18.09 (25.68)		43.06 (46.31)	
Layoffs (*n* = 12)	72.25 (52.10)		24.67 (19.31)		47.58 (37.82)	
Lost Job (*n* = 10)	75.80 (74.14)		18.10 (25.08)		57.70 (50.96)	
Child Age						
4–6 (*n* = 69)	55.32 (55.34)	F = 0.999 *p* = 0.39 *ηp*^2^ = 0.011	14.20 (20.34)	F = 1.45 *p* = 0.22 *ηp*^2^ = 0.016	41.12 (37.80)	F = 0.810 *p* = 0.48 *ηp*^2^ = 0.009
7–10 (*n* = 93)	59.52 (58.84)		18.35 (22.36)		41.15 (40.27)	
11–13 (*n* = 64)	56.47 (63.98)		17.36 (23.81)		39.11 (42.38)	
14–17 (*n* = 52)	73.06 (68.11)		22.88 (25.45)		50.17 (44.69)	
Child Sex						
Boys (*n* = 156)	57.78 (63.39)	F = 0.605 *p* = 0.43 *ηp*^2^ = 0.002	16.34 (23.79)	F = 1.74 *p* = 0.18 *ηp*^2^ = 0.006	41.44 (42.38)	F = 0.177 *p* = 0.67 *ηp*^2^ = 0.001
Girls (*n* = 122)	63.52 (58.02)		19.99 (21.61)		43.53 (39.29)	
Child Atypical Development						
Yes (*n* = 38)	33.55 (68.62)	F = 8.68 *p* = 0.003 * ηp^2^ = 0.031	6.68 (27.12)	F = 11.03 *p* = 0.001 ** ηp^2^ = 0.038	26.87 (44.54)	F = 6.408 *p* = 0.01 * ηp^2^ = 0.023
Not (*n* = 240)	64.54 (58.81)		19.72 (21.68)		44.81 (39.95)	

Note: *: *p* < 0.05; **: *p* < 0.001.

**Table 3:** Correlation between BR2, neuroticism personality factor, and parental competence variables.

Variables	M (SD)	1	2	3	4	5	6	7
1. BR^2^ Tot	60.30 (61.05)	-						
2. Common Antecedents	17.94 (22.89)	0.920 **	-					
3. Specific Antecedents	42.36 (40.99)	0.976 **	0.811 **	-				
4. Neuroticism	9.65 (2.95)	−0.281 **	−0.389 **	−0.201 **	-			
5. Parental Competence	60.31 (10.83)	0.349 **	0.299 **	0.353 **	−0.209 **	-		
6. Satisfaction	34.26 (7.48)	0.313 **	0.291 **	0.303 **	−0.197 **	0.866 **	-	
7. Efficacy	26.05 (5.73)	0.252 **	0.184 **	0.272 **	−0.138 *	0.758 **	0.331 **	-

Note: *: *p* < 0.05; **: *p* < 0.01.

## Data Availability

The data presented in this study are available on request from the 495 corresponding author.
